# Fibrillary Glomerulonephritis in a Patient with Sjogren’s Syndrome

**DOI:** 10.7759/cureus.2483

**Published:** 2018-04-15

**Authors:** Muhammad Uzair Lodhi, Tahira Sabeen Saleem, Muhammad Shariq Usman, Waliul Chowdhury, Aaron R Kuzel, Hafiz Imran Iqbal, Mustafa Rahim

**Affiliations:** 1 Medical Student, Department of Medicine, Raleigh General Hospital, Beckley, Wv; 2 Department of Medicine, Raleigh General Hospital, Beckley, Wv; 3 Internal Medicine, Civil Hospital Karachi, Dow University of Health Sciences, Karachi, Pakistan; 4 Department of Emergency Medicine, Lincoln Memorial University-Debusk College of Osteopathic Medicine; 5 Nephrologist, Department of Medicine, Raleigh General Hospital, Beckley, Wv; 6 Assistant Clinical Professor of Internal Medicine, West Virginia University School of Medicine

**Keywords:** fibrillary glomerulonephritis, non-amyloid fibrillary deposits, sjogren's syndrome, electron microscopy, amyloidosis vs fibrillary glomerulonephritis, fibrillary glomerulonephritis vs immunotactoid disease, negative congo red staining

## Abstract

Fibrillary glomerulonephritis (FGN) is an uncommon cause of primary glomerular disease. FGN is usually idiopathic; however, it has been associated with underlying malignancy or autoimmune diseases in some patients as well. The most commonly found autoimmune diseases in FGN patients include Graves’ disease, systemic lupus nephritis, Chron’s disease, and idiopathic thrombocytopenia purpura. FGN in a patient with underlying asymptomatic Sjogren’s syndrome is very rare in the literature, with only two previously reported cases of this association. We present the case of a 75-year-old female with a past medical history of asymptomatic primary Sjogren's syndrome and fibromyalgia, who presented to emergency department with a new episode of hypertension. The electron microscopy (EM) showed randomly arranged nonamyloid fibrillar deposits in the mesangium and glomerular capillary walls, confirming FGN. In this case-based review, we describe in detail the diagnostic work-up, clinical course, and complications in management. We also discuss some of the other nonamyloid fibrillary glomerular diseases.

## Introduction

Fibrillary glomerulonephritis (FGN) occurs in about 0.5% to 1% of all nontransplant renal biopsies [1-2]. It usually presents with nephrotic range proteinuria, hypertension, hematuria, and renal insufficiency. The diagnosis of FGN is established by renal biopsy, with electron microscopy (EM) showing the pathognomonic histologic findings of randomly arranged fibrillar deposits in the mesangium and glomerular basement membrane [1,3]. The fibrils in FGN are 16-24 nanometer (nm) in size, and they do not stain with Congo red [1]. FGN carries a poor prognosis, with almost 50% of the patients progressing to end-stage renal disease within three years of diagnosis [1, 3-4].

## Case presentation

History and physical examination

A 75-year-old female with a past medical history of fibromyalgia and Sjogren’s syndrome presented to the emergency department with severe hypertension. The patient was not taking any medication at home. On physical examination, the patient was in slight distress. The vitals were as follows: blood pressure of 182/91 mmHg, heart rate of 72 beats per minute, respiratory rate of 15 breaths per minute, and oxygen saturation of 93% on room air. The patient had 2+ pitting edema of lower extremities. The rest of the physical examination was unremarkable.

Hospital course

The blood pressure of the patient improved after giving hydralazine and clonidine. Nephrology was consulted for evaluation of increased creatinine (3.8 mg/dl) with blood urea nitrogen of 29 mg/dl. Review of the past medical record in the hospital showed her creatinine level of 2.5 mg/dl, a year ago. Further workup revealed 24-hour proteinuria of 3.89 grams. Urine dipstick showed 3+ protein, 5-10 red blood cells and 10-15 white blood cells per high power field. Considering the past medical history of Sjogren’s syndrome, the patient was suspected to have acute interstitial nephritis or possible immune-complex-mediated disease. Her C3 and C4 complement levels were low. Serum immunofixation did not reveal any monoclonal immunoglobulin. Serologies for antinuclear antibody was positive (titer, 1:640), negative for anti-double-stranded DNA, hepatitis B and C, and antineutrophilic cytoplasmic antibodies.

On light microscopy (LM), three glomeruli were present for evaluation, two of which were globally sclerotic and one showed segmental scarring. The segmental lesion had an accompanying fibrous reaction, suggestive of a possible healed/fibrous crescent. The glomeruli also featured noticeable mesangial expansion, which was negative for Silver methenamine and positive for Periodic acid-Schiff (PAS) staining. Congo-red stain was also negative. No capillary wall deposits were seen on special stains and no active necrotizing or crescent lesions were present. Moderate interstitial fibrosis was present in the interstitium. Furthermore, moderate intimal fibrosis was seen in vessels, with no thrombosis or vasculitis.

**Figure 1:**
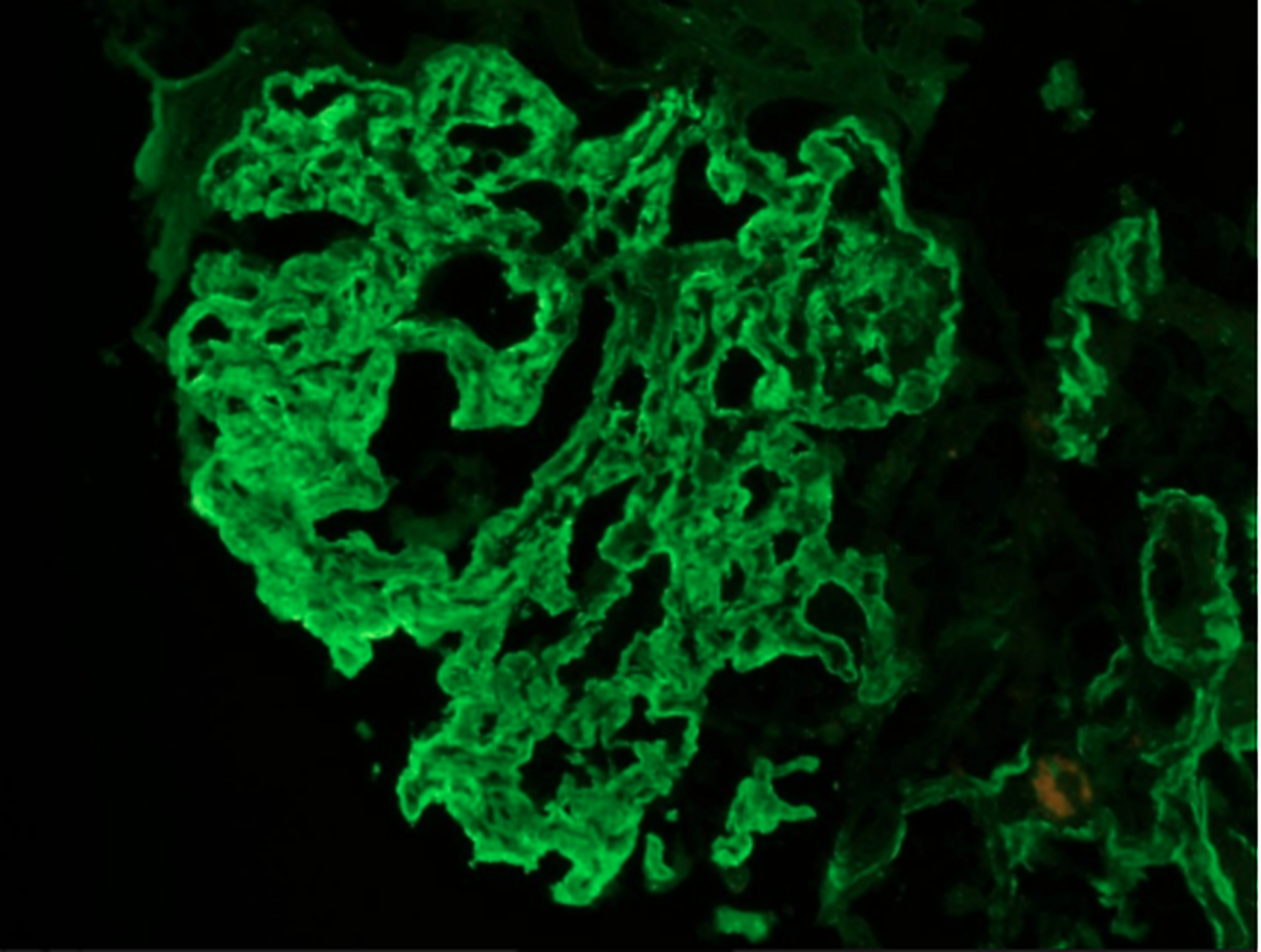
Immunofluorescence image of the patient showing positive staining with immunoglobulin G (IgG)

**Figure 2:**
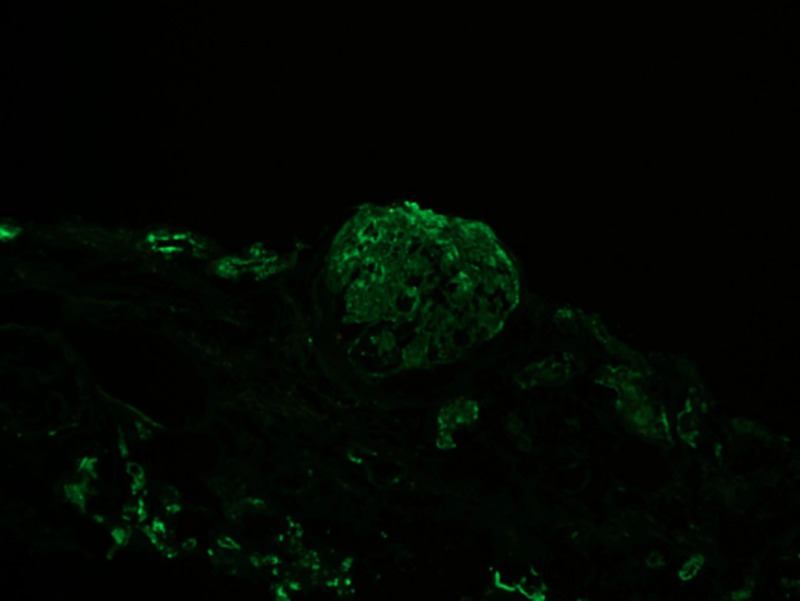
Immunofluorescence image of the patient showing 2+ staining for C3

Immunofluorescent (IF) showed diffuse global 3+ smudgy mesangial; the capillary wall was positivity noted with immunoglobulin G (IgG) (Figure 1). Glomerular staining showed 1+ IgM, C1q, 2+ C3, 1+ staining with both kappa and lambda light chains (Figure 2). IgG and albumin stained protein reabsorption granules in the tubular cytoplasm were noted. No significant staining was seen with fibrinogen.

**Figure 3:**
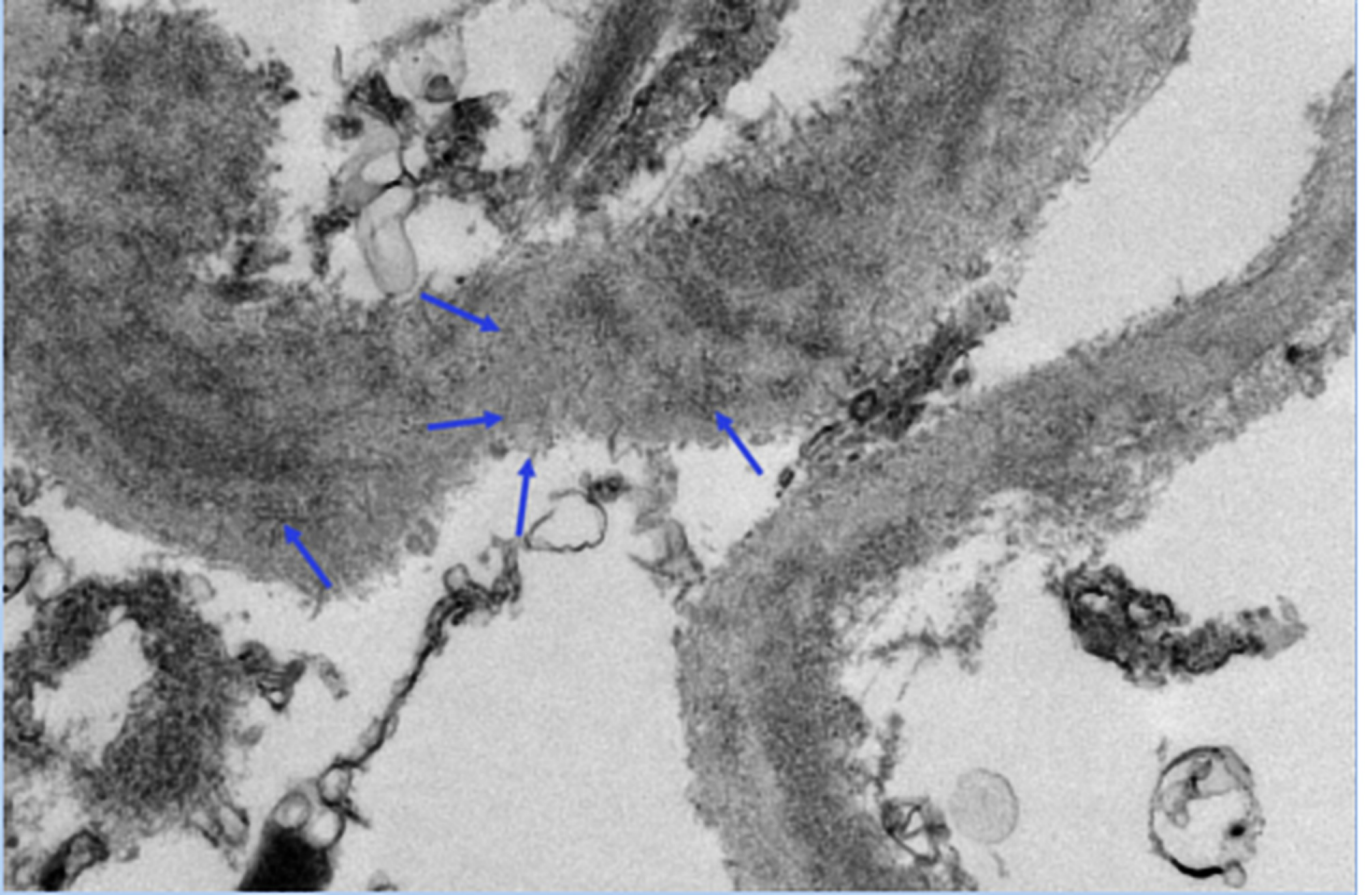
Electron microscopy of the patient showing fibrillary deposition (blue arrows) in the mesangium

**Figure 4:**
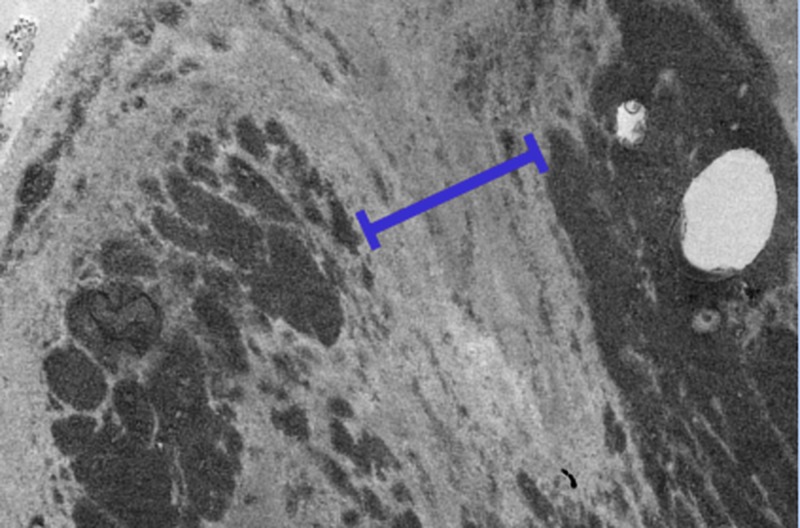
Electron microscopy of the patient showing thickened mesangium (blue mark)

EM confirmed the presence of extensive electron dense deposits in the expanded mesangial regions and throughout the thickened glomerular basement membranes (Figures 3-4). These deposits had a distinct fibrillary substructure and they did not show any transmembranous spicule formation. No extra-glomerular deposits were seen.

## Discussion

Differential diagnosis

The algorithmic approach to glomerular disease with organized deposits starts off with Silver methenamine staining. The disorders (collagenofibrotic glomerulopathy) that result in the expansion of mesangial extracellular matrix stain positive with Silver methenamine. On the other hand, the disorders that replace the normal mesangial matrix do not stain with Silver methenamine. These Silver methenamine negative disorders have to further go through Congo red staining. The Congo red positive depositions are characterized as amyloidosis. However, the Congo red negative (non amyloid) depositions have to be further divided based on whether the fibrils are immunoglobulin derived (as in immunotactoid glomerulopathy (ITG), FGN) or non-immunoglobulin derived (as in fibronection glomerulopathy) [5]. The pathognomonic findings on EM were used to make a definitive diagnosis of FGN, and to differentiate it from amyloidosis as well as ITG [5].

FGN Versus Amyloidosis

Both FGN and amyloidosis do not stain with Silver methenamine [5]. Unlike amyloid fibrils, the fibrils in FGN do not stain with Congo red, serum amyloid A, thioflavine-T, or with antibodies to a specific light chain [6-8]. Additionally, on EM, the FGN presents as random fibrils of 16-24 nm in size, while amyloidosis presents as non-branching fibrils of 8-12 nm in size [1-4].

FGN Versus Immunotactoid Disease

As FGN is characterized by the pathognomonic finding of fibrils of 16-24 nm; the immunotactoid disease is characterized by the deposition of microtubules 30-50 nm in size [2-3,7,9]. Apart from the differences on EM, FGN and ITG share many similarities. Both FGN and ITG do not stain with Silver methenamine [5]. The similarities in the clinical presentation, associated conditions, and prognosis have led many experts to debate on whether FGN and ITG are same or distinct disease entities [3].

*FGN Versus Fibronectin Glomerulopathy* FGN and fibronectin glomerulopathy both stain negative with Silver methenamine as well as Congo red [5]. However, the fibrils in FGN are immunoglobulin derived, while the fibrils in fibronectin glomerulopathy are non-immunoglobulin derived. Furthermore, the fibrils in fibronectin are much smaller than the fibrils in FGN [5].

Treatment

FGN has an unknown pathogenesis. The rarity of this disease, combined with lack of standardized treatment has resulted in poor prognosis. Immunosuppressive therapy consisting of glucocorticoids with or without other agents (such as mycophenolate, cyclophosphamide, cyclosporine, and rituximab) has shown inconsistent results in some uncontrolled studies [1,3,8-11].

Our patient was not able to withstand the immunosuppressant treatment due to her fragile state. She was continued on glucocorticoids and angiotensin-converting-enzyme inhibitors. The renal function of the patient improved for about a year as her 24-hour proteinuria decreased to less than one gram after the initiation of the treatment; however, it suddenly started to get worse. The patient developed hyperkalemia and bicarbonate-resistant metabolic acidosis. Since then she has been on hemodialysis.

The recurrence of disease after the renal transplantation is another dilemma, which researchers have to address [12-14]. A study conducted by Nasr et al. in 2011, reported a biopsy-proven disease recurrence in 36% of the total 14 patients who had kidney transplant [8].

As one-third of the FGN patients have malignancy, monoclonal gammopathy, and autoimmune diseases, treatment of the underlying disease in these patients may hinder the progression of FGN.

Literature review

Our review of the literature revealed two previous cases of FGN secondary to Sjogren’s syndrome. In one of the cases, a 77-year-old male with biopsy-proven FGN was started on prednisone and azathioprine; however, the patient ended up requiring hemodialysis within four weeks of diagnosis. On a one-year follow-up, the patient was fairly stable on hemodialysis [15]. The age, treatment, and prognosis of the second reported case of FGN secondary to Sjogren’s disease, is unknown [8].

Recent developments

While there is a lack of randomized trials being currently performed to establish standardized treatment for the patients with FGN, there has been a remarkable discovery towards improving the rapid diagnosis and understanding the pathogenesis of the FGN. In 2018, a study by Dasari et al. detected DnaJ heat shock protein family B member 9 (DNAJB9) as one of the most abundant proteins in FGN glomeruli. No staining was observed in patients with amyloidosis, other glomerular diseases (such as ITG), or healthy controls. This is why the study proposed DNAJB9 to have 100% sensitivity and specificity for FGN [16]. Due to a very high sensitivity and specificity of DNAJB9 for FGN, another study in 2018 by Andeen et al. also identified DNAJB9 as a possible autoantigen in FGN, and suggested IgG1 and classic complement effector pathways as likely mediators of the glomerular injury in FGN [17].

In addition to giving us a better understanding of the pathogenesis behind FGN, these two proteomic studies also suggest that detection of DNAJB9 by immunofluorescence microscopy could establish the diagnosis of FGN without the need for electron microscopy.

## Conclusions

Even though some noticeable advancements have recently begun to investigate the pathogenesis of FGN, as of now it still remains a mystery. Further studies are required to confirm the role of DNAJB9 in the pathogenesis of FGN. Controlled clinical trials are also needed to evaluate the effect of immunosuppressant treatments. Additionally, due to the rarity of this disease and lack of literature, physicians should carefully look for any underlying condition in a patient with FGN, as treating the underlying conditions may help us in slowing the progression of this disease.
